# Overview and Methods for the Youth Risk Behavior Surveillance System — United States, 2021

**DOI:** 10.15585/mmwr.su7201a1

**Published:** 2023-04-28

**Authors:** Jonetta J. Mpofu, J. Michael Underwood, Jemekia E. Thornton, Nancy D. Brener, Adriana Rico, Greta Kilmer, William A. Harris, Michelle Leon-Nguyen, David Chyen, Connie Lim, Cecily K. Mbaka, Jennifer Smith-Grant, Lisa Whittle, Sherry Everett Jones, Kathleen H. Krause, Jingjing Li, Shari L. Shanklin, Izzy McKinnon, Loredona Arrey, Barbara E. Queen, Alice M. Roberts

**Affiliations:** ^1^Division of Adolescent and School Health, National Center for HIV, Viral Hepatitis, STD, and TB Prevention, CDC, Atlanta, Georgia; ^2^U.S. Public Health Service Commissioned Corps, Rockville, Maryland; ^3^Westat, Rockville, Maryland; ^4^ICF International, Rockville, Maryland

## Abstract

The Youth Risk Behavior Surveillance System (YRBSS) is the largest public health surveillance system in the United States, monitoring a broad range of health-related behaviors among high school students. The system includes a nationally representative Youth Risk Behavior Survey (YRBS) and separate school-based YRBSs conducted by states, tribes, territories, and local school districts. In 2021, these surveys were conducted during the COVID-19 pandemic. The pandemic underscored the importance of data in understanding changes in youth risk behaviors and addressing the multifaceted public health needs of youths. This overview report describes 2021 YRBSS survey methodology, including sampling, data collection procedures, response rates, data processing, weighting, and analyses. The 2021 YRBS participation map, survey response rates, and a detailed examination of student demographic characteristics are included in this report. During 2021, in addition to the national YRBS, a total of 78 surveys were administered to high school students across the United States, representing the national population, 45 states, two tribal governments, three territories, and 28 local school districts. YRBSS data from 2021 provided the first opportunity since the onset of the COVID-19 pandemic to compare youth health behaviors using long-term public health surveillance. Approximately half of all student respondents represented racial and ethnic minority groups, and approximately one in four identified as lesbian, gay, bisexual, questioning, or other (a sexual identity other than heterosexual) (LGBQ+). These findings reflect shifts in youth demographics, with increased percentages of racial and ethnic minority and LGBQ+ youths compared with previous YRBSS cycles. Educators, parents, local decision makers, and other partners use YRBSS data to monitor health behavior trends, guide school health programs, and develop local and state policy. These and future data can be used in developing health equity strategies to address long-term disparities so that all youths can thrive in safe and supportive environments. This overview and methods report is one of 11 featured in this *MMWR* supplement. Each report is based on data collected using methods presented in this overview. A full description of YRBSS results and downloadable data are available (https://www.cdc.gov/healthyyouth/data/yrbs/index.htm).

## Introduction

The COVID-19 pandemic has affected the mental, physical, and emotional health of adolescents (*1*,*2*). Adolescents have endured the stress of the pandemic, resulting social distancing measures, and societal discord in ways unique to their young age. Trends in adolescent behavioral and emotional health that were of concern before the pandemic have worsened (*3*,*4*). The pandemic (including the loss of life, disease burden, and measures to counteract its effects) exposed structural, social, and economic conditions that accelerated certain health behaviors among students and worsened existing health disparities for racial and ethnic and sexual minority youths (*2*). During January–June 2021, CDC administered the nationally representative Adolescent Behaviors and Experiences Survey (ABES) to assess student health behaviors and experiences during the pandemic (*5*). Results from ABES indicated that 37% of high school students experienced poor mental health during the pandemic and 44% felt persistently sad or hopeless during the previous 12 months, a period covered by the World Health Organization’s pandemic declaration on March 11, 2020 (*3*). Approximately one in three youths have ever experienced racism at school, which was significantly associated with poor mental health (*6*). Students who described their sexual identity as lesbian, gay, bisexual, questioning, or other (a sexual identity other than heterosexual) (LGBQ+) had a higher prevalence of poor mental health during the pandemic than heterosexual students (*3*). Approximately half of students reported experiencing emotional abuse by a parent or another adult in their home (55%), with the highest prevalence among non-Hispanic multiracial students (66%) and students identifying as questioning or other (76%) (*7*). In addition, although approximately one fourth of all students experienced hunger during the pandemic, White students experienced significantly less hunger (18.5%) than all other racial and ethnic groups (*7*).

The pandemic’s effect on student health behaviors cannot be separated from the context of changing youth demographics in the United States. After years of monitoring trends in demographic data (*8*), White youths no longer represent the majority of students enrolled in K–12 public schools in the United States (*9*). Nationally representative 2017 and 2019 Youth Risk Behavior Survey (YRBS) data, and most recently ABES data, have demonstrated steady increases in the proportion of students who identify as lesbian, gay, or bisexual (10.4%, 11.2%, and 15.3%, respectively) (*5*,*10*,*11*). The shifting demographics of adolescents in the United States and the health disparities faced by youths from racial and ethnic and sexual minority groups make gathering information on their health behaviors and associated protective factors more important than ever.

Since 1991, the Youth Risk Behavior Surveillance System (YRBSS) has monitored health behaviors, experiences, and conditions affecting the health outcomes of high school students in the United States. The system is composed of a national school-based survey administered by CDC and multiple site-level, school-based surveys administered by states, tribal governments, territories, and local school districts. YRBSS offers a unique opportunity to monitor the prevalence of important youth health behaviors and long-term trends on certain risk behaviors, including those that have been monitored for more than 20 years. The 2021 YRBSS captured information on the following topics: student demographics (sex, sexual identity, race and ethnicity, and grade), youth health behaviors and conditions (sexual; injury and violence; bullying; diet and physical activity; obesity; and mental health, including suicide), and substance use behaviors (electronic vapor product and tobacco product use, alcohol use, and other drug use). Changes to the national questionnaire in 2021 included new questions that examined urgent and relevant student health behaviors and experiences, including protective factors (parental monitoring and school connectedness), housing instability, exposure to community violence, and mental health during the COVID-19 pandemic. Data from the 2021 survey administration offered the first direct comparison with behaviors before onset of the COVID-19 pandemic.

This report describes the 2021 YRBSS methodology, including sampling, data collection, processing, weighting, and analyses. This overview and methods report is one of 11 included in this *MMWR* supplement featuring 2021 national YRBS data. Each of the other 10 reports uses 2021 YRBS data to assess a priority health topic for adolescents (*12*). This supplement does not include data from site-level surveys; however, those results can be found in YRBS Explorer (https://yrbs-explorer.services.cdc.gov), CDC’s web-based application for YRBSS data. Topic areas of focus in this supplement include updates regarding electronic vapor product use, dietary behaviors and physical activity, and interpersonal violence and new national data on housing instability, exposure to community violence, school connectedness, and parental monitoring. Public health practitioners and researchers can use YRBSS data to examine the prevalence of youth health risk behaviors, monitor trends, and guide interventions.

## National YRBS Methodology

### Overview

The national YRBS is conducted biennially, typically during the spring (January–June) of odd-numbered years among students in grades 9–12 enrolled in U.S. public and private schools. However, the 2021 national YRBS administration was postponed until fall (September–December) 2021 because of the COVID-19 pandemic and the shift to virtual and hybrid school instructional models and ongoing school closures during spring 2021. Biennial administration of the YRBS allows CDC to assess temporal changes in risk behaviors among the U.S. high school population. The national YRBS provides comparable data across survey years and allows state and local entities that conduct their own YRBSs to compare risk behaviors of their youths with those at the national level. A nationally representative sample of schools and a random sample of classes within those schools are selected to participate.

### Questionnaire

In 2021, the national YRBS questionnaire consisted of 99 questions. Of those, 87 questions were included in the standard questionnaire* used by sites. Twelve questions were added to the standard questionnaire that reflected areas of interest for CDC and other partners. As in all cycles, both the previous year’s standard questionnaire and additional national-only questions were revised to include measurement of emerging and prevailing risk behaviors among high school students. Subject matter experts from CDC and elsewhere proposed changes, additions, and deletions to the questionnaire. Further refinements to the questionnaire were made based on feedback from cognitive testing. During this process the sexual identity question was modified (Table 1). In addition, in 2021, the national YRBS questionnaire was offered for the first time in English and Spanish.

**TABLE 1:** Sexual identity and sexual contact questions on the Youth Risk Behavior Survey — United States, 2021

Question	Student response		Description for analysis
**Sexual identity**			
**Which of the following best describes you?** 1) Heterosexual (straight), 2) gay or lesbian, 3) bisexual, 4) I describe my sexual identity some other way, 5) I am not sure about my sexual identity (questioning), or 6) I do not know what this question is asking	Heterosexual (straight) (1)		Heterosexual students (1)
	Gay or lesbian (2) or bisexual (3)		Lesbian, gay, or bisexual students (2 or 3)
	Other (4) or questioning (5)		Other or questioning students (4 or 5)
	Did not understand (6)		Students missing sexual identity variable (6)
**Sex of sexual contacts**			
**During your life, with whom have you had sexual contact?** 1) I have never had sexual contact, 2) females, 3) males, or 4) females and males What is your sex? 1) Female, or 2) male	I have never had sexual contact*		Students who had no sexual contact
	**Contact:** Female Male	**Student:** Male Female	Students who had sexual contact with only the opposite sex
	**Contact:** Male Females and males Female Females and males	**Student:** Male^§^ Male Female^†,§^ Female	Students who had sexual contact with only the same sex or with both sexes

* Excluded from analyses on sexual behaviors.

^†^ Excluded from analyses on condom use.

^§^ Excluded from analyses on dual use of condoms and birth control.

All questions, except those assessing height, weight, and race, were multiple choice, with a maximum of eight mutually exclusive response options and only one possible answer per question. Most of the 2021 survey questions underwent test-retest analysis and demonstrated good reliability (*13*,*14*). The wording of each question, including recall periods, response options, and operational definitions for each variable, are available in the 2021 YRBS questionnaire and data user’s guide. (YRBSS data and documentation are available at https://www.cdc.gov/healthyyouth/data/yrbs/data.htm.)

In accordance with guidance from subject matter experts, response options for the sexual identity question were updated for the 2021 YRBS to include the following new categories: “I am not sure about my sexual identity (questioning),” “I describe my sexual identity in some other way,” and “I do not know what this question is asking.” As a result, beginning in 2021, YRBS can provide data for LGBQ+ students, as opposed to only lesbian, gay, or bisexual students.

### Sampling

The 2021 YRBS sampling frame consisted of all regular public schools (including charter schools), parochial schools, and other private schools with students in at least one of grades 9–12 in the 50 U.S. states and the District of Columbia. Alternative schools, special education schools, schools operated by the U.S. Department of Defense or the Bureau of Indian Education, and vocational schools serving students who also attended another school were excluded. Schools with ≤40 students enrolled in grades 9–12 also were excluded. The sampling frame was constructed from data files obtained from MDR (formerly Market Data Retrieval) and the National Center for Education Statistics (NCES). NCES data sources included the Common Core of Data (https://nces.ed.gov/ccd) for public schools and the Private School Survey (https://nces.ed.gov/surveys/pss) for private schools. The YRBS sample size was increased in 2021 in anticipation of lower response rates resulting from the COVID-19 pandemic and to obtain a large enough sample size for the desired precision.

A three-stage cluster sampling design was used to produce a nationally representative sample of students in grades 9–12 who attend public and private schools. The first-stage sampling frame comprised 1,257 primary sampling units (PSUs), which consisted of entire counties, groups of smaller adjacent counties, or parts of larger counties. PSUs were categorized into 16 strata according to their metropolitan statistical area status (i.e., urban or nonurban) and the percentages of Black and Hispanic or Latino (Hispanic) students in each PSU. Sixty of the 1,257 PSUs were sampled with probability proportional to overall school enrollment size for that PSU. For the second-stage sampling, secondary sampling units (SSUs) were defined as a physical school with grades 9–12 or a school created by combining nearby schools to provide all four grades. From the 60 PSUs, 180 SSUs were sampled with probability proportional to school enrollment size. To provide adequate coverage of students in small schools, an additional 20 small SSUs were selected from a subsample of 20 of the 60 PSUs. These 200 SSUs corresponded to 209 physical schools. The third stage of sampling comprised random sampling of one or two classrooms in each of grades 9–12 from either a required subject (e.g., English or social studies) or a required period (e.g., homeroom or second period). All students in sampled classes who could independently complete the survey were eligible to participate. Schools, classes, and students that refused to participate were not replaced.

### Data Collection Procedures

Institutional review boards at CDC and ICF, the survey contractor, approved the protocol for the YRBS. Data collection was conducted consistent with applicable federal law and CDC policy.^†^ Survey procedures were designed to protect students’ privacy by allowing for anonymous participation. Participation was voluntary, and local parental permission procedures were followed before survey administration. During survey administration, students completed the self-administered questionnaire during one class period (approximately 45 minutes) and recorded their responses on a computer-scannable booklet.

### Response Rates and Data Processing

For the 2021 YRBS, 17,508 questionnaires were completed in 152 schools. The national data set was cleaned and edited for inconsistencies. Missing data were not statistically imputed. A questionnaire failed quality control when <20 responses remained after editing or when it contained the same answer to ≥15 consecutive questions. Among the 17,508 completed questionnaires, 276 failed quality control and were excluded from analysis, resulting in 17,232 usable questionnaires. The school response rate was 72.7%, the student response rate was 79.1%, and the overall response rate (i.e., [student response rate] x [school response rate]) was 57.5%.

Race and ethnicity were ascertained from two questions: 1) “Are you Hispanic or Latino?” (with response options of “yes” or “no”) and 2) “What is your race?” (with response options of “American Indian or Alaska Native [AI/AN],” “Asian,” “Black or African American [Black],” “Native Hawaiian or other Pacific Islander [NH/OPI],” or “White”). (Persons of Hispanic or Latino [Hispanic] origin might be of any race but are categorized as Hispanic; all racial groups are non-Hispanic.) For the second question, students could select more than one response option. For this report, students were classified as Hispanic or Latino and are referred to as Hispanic if they answered “yes” to the first question, regardless of how they answered the second question. For example, students who answered “no” to the first question and selected only Black or African American to the second question were classified as Black or African American and are referred to as Black. Likewise, students who answered “no” to the first question and selected only White to the second question were classified and are referred to as White. Race and ethnicity were classified as missing for students who did not answer the first question and for students who answered “no” to the first question but did not answer the second question. Students who selected more than one response option to “What is your race?” were classified as multiracial. Further, to meet the needs of an increasingly diverse population, CDC implemented modified suppression criteria for the YRBSS in 2021, allowing for increased data representation from students of diverse racial and ethnic groups. Previously, estimates with a denominator of <100 were suppressed; however, many of these estimates were found to be statistically reliable according to criteria set forth by CDC’s National Center for Health Statistics (*15*). Guided by these criteria, and in consideration of criteria used for other national surveillance systems, YRBS estimates with a denominator of <30 were suppressed in all years.

To obtain a sufficient sample size for analyses of health-related behaviors by sexual orientation (sexual identity and sex of sexual contacts), students were divided into groups (Table 1). Students who had no sexual contact were excluded from analyses related to sexual behaviors. Female students who had sexual contact with only females were excluded from analyses on condom use and dual use of condoms and birth control, and male students who had sexual contact with only males were excluded from analyses on dual use of condoms and birth control.

### Weighting

A weight based on student sex, race and ethnicity, and grade was applied to each record to adjust for school and student nonresponse and oversampling of Black and Hispanic students. The overall weights were scaled so that the weighted count of students equals the total sample size, and the weighted proportions of students in each grade match the national population proportions. Therefore, weighted estimates are nationally representative of all students in grades 9–12 attending U.S. public and nonpublic schools.

### Analytic Methods

Findings presented in this *MMWR* supplement are derived from analytic procedures similar to what is described in this overview report. For more information regarding the detailed analyses presented in this supplement (e.g., variables analyzed, custom measures, and data years), see the methods section in each individual report.

All statistical analyses were conducted using SAS-callable SUDAAN (version 11.0.3; RTI International) to account for the complex sampling design and weighting. In all reports, prevalence estimates and CIs were computed for variables used in those reports. Prevalence estimates where the denominator was <30 were suppressed. Pairwise differences between groups (e.g., sex, race and ethnicity, grade, sexual identity, and sex of sexual contacts) were determined using *t*-tests with Taylor series linearization. Pairwise differences were considered statistically significant if the *t*-test p value was <0.05. Chi-square tests were used to examine comparisons between risk behaviors and experiences by demographic and behavioral characteristics (race and ethnicity, grade, sexual identity, and sex of sexual contacts). Chi-square tests were considered statistically significant if the p value was <0.05.

In reports that analyzed temporal trends, logistic regression analyses were used to examine linear and quadratic changes in estimates, controlling for sex, grade, and racial and ethnic changes over time. A p value of <0.05 associated with a regression coefficient was considered statistically significant. Linear and quadratic time variables were treated as continuous and were coded by using orthogonal coefficients calculated with PROC IML in SAS (version 9.4; SAS Institute). A minimum of 3 survey years was required for calculating linear trends, and a minimum of 6 survey years was required to calculate quadratic trends. Separate regression models were used to assess linear and quadratic trends. When a significant quadratic trend was identified, Joinpoint (version 4.9; National Cancer Institute) was used to automate identification of the year when the trend changed. Regression models were used to identify linear trends occurring before and after the change in trend. A quadratic trend indicates a statistically significant but nonlinear change in prevalence over time. A long-term temporal change that includes a significant linear and quadratic trend demonstrates nonlinear variation (e.g., leveling off or change in direction) in addition to an overall increase or decrease over time. Cubic and higher-order trends were not assessed.

In reports that analyzed 2-year changes in health-related behaviors, prevalence estimates from 2019 and 2021 were compared by using *t*-tests for variables assessed with identically worded questions in both survey years. An exception was made for birth control use, where the wording specifically addressed sexual contact with opposite sex partners in 2021 but not in 2019. Prevalence estimates were considered statistically different if the *t*-test p value was <0.05. For 2-year changes assessed with absolute measures (i.e., prevalence difference), 95% CIs that did not cross zero were considered statistically significant. For relative measures (i.e., prevalence ratio), 95% CIs that did not cross 1.0 were considered statistically significant.

### Data Availability and Dissemination

National and site-level YRBS data (1991–2021) are available in a combined data set from the YRBSS data and documentation website (https://www.cdc.gov/healthyyouth/data/yrbs/data.htm), as are additional resources, including data documentation and analysis guides. Data are available in both Access and ASCII formats, and SAS and SPSS programs are provided for converting the ASCII data into SAS and SPSS data sets. Variables are standardized to facilitate trend analyses and for combining data. YRBSS data also are available online via three web-based data dissemination tools: Youth Online, YRBS Analysis Tool, and YRBS Explorer. Youth Online allows point-and-click data analysis and creation of customized tables, graphs, maps, and fact sheets (https://nccd.cdc.gov/Youthonline/App/Default.aspx). Youth Online also performs statistical tests by health topic and filters and sorts data by race and ethnicity, sex, grade, and sexual orientation. The YRBS Analysis Tool allows real-time data analysis of YRBS data that generates frequencies, cross-tabulations, and stratified results (https://nccd.cdc.gov/YRBSSanalysis). YRBS Explorer is an application featuring options to view and compare national, state, and local data via tables and graphs (https://yrbs-explorer.services.cdc.gov). Data requests and other YRBSS-related questions can be sent to CDC by using the data request form (https://www.cdc.gov/healthyyouth/data/yrbs/contact.htm).

## State, Tribal, Territorial, and Local School District YRBS Methodology

### Overview

**FIGURE 1:**
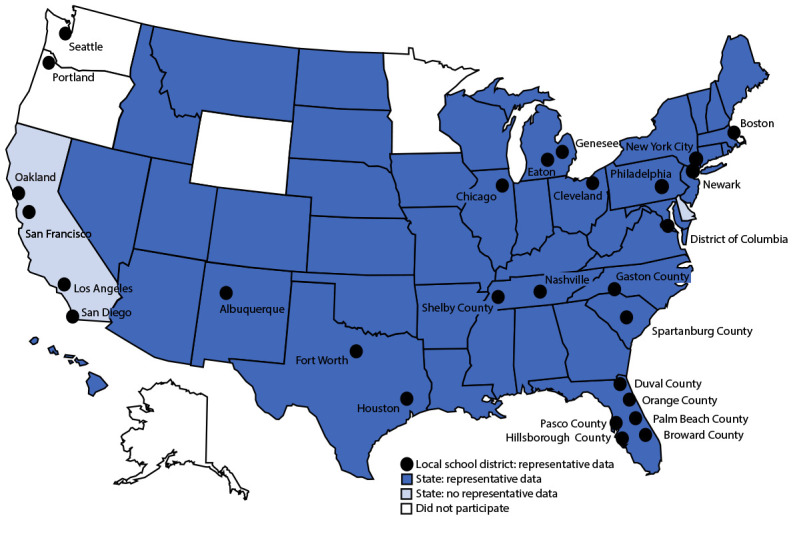
State, tribal government, territorial, and local school district Youth Risk Behavior Surveys — selected U.S. sites, 2021

**FIGURE 2:**
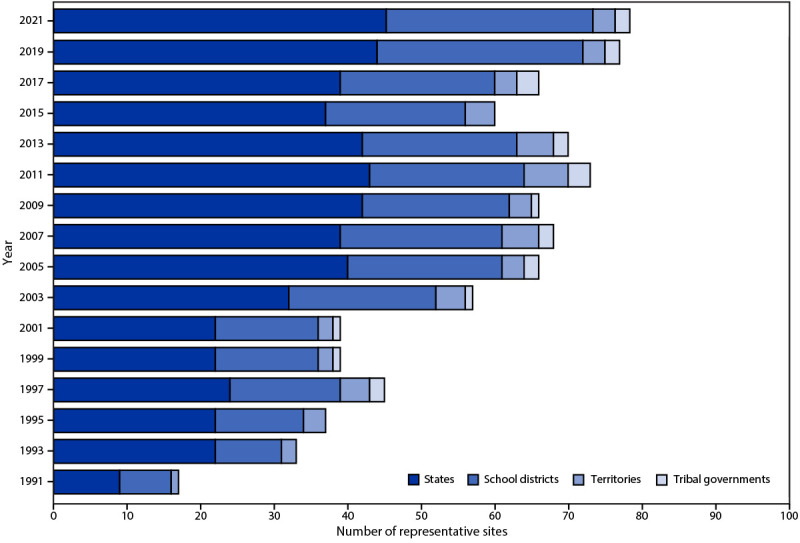
Number of states, tribal governments, territories, and local school districts with representative Youth Risk Behavior Survey data, by year of survey — selected U.S. sites, 1991–2021

Biennial administration of site-level YRBSs allows state and local education and health agencies to assess how risk behaviors temporally change among the high school populations in their respective jurisdictions. Site-level YRBS data provide comparable data across years and allow comparisons of student behaviors across jurisdictions (e.g., national to state). Site-level surveys are conducted among students in grades 9–12 attending public schools by using samples representative of the state, tribal, territorial, or local jurisdiction where they are administered. Seventy-eight sites administered a YRBS in 2021 (45 states, two tribal governments, three territories, and 28 local school districts) (Figures 1 and 2). Sites^§^ administered their surveys during spring (nine sites) or fall 2021 (69 sites). The survey is self-administered anonymously and takes one class period (approximately 45 minutes) to complete. State and local institutional review boards approved the protocol for their respective YRBSs. Survey methodology for data collection, processing, and analytic methods were the same as those described for the YRBS; however, 29 sites collected data electronically using computers, smartphones, or tablets.

### Questionnaires

The 2021 YRBS standard questionnaire contained 87 questions and was used as the starting point for site-level YRBS questionnaires. Sites could add or delete questions but were required to use at least 58 of the questions on the standard questionnaire. This flexibility allowed YRBS coordinators and other local partners the opportunity to pursue topics of interest by customizing their survey.

### Sampling

Sites used a two-stage cluster sampling design to produce a representative sample of students in grades 9–12 in their jurisdiction. In 43 states, one tribe, one territory, and four local school districts, in the first sampling stage, public schools with any of grades 9–12 were sampled with probability proportional to school enrollment size. In two states and 24 local school districts, all schools in the jurisdiction were selected to participate (i.e., a census of schools). In the second sampling stage, intact classes from either a required subject (e.g., English or social studies) or a required period (e.g., homeroom or second period) were sampled randomly. In three sites (Vermont, the District of Columbia, and the Winnebago Tribe of Nebraska), a census of students was selected to participate. All students in selected classes who could independently complete the survey were eligible to participate.

Students in schools that were in both the national sample and a site-level sample were asked to participate in only one survey. Either the national questionnaire or the state or local school district’s YRBS questionnaire was administered to the students in these schools, and data from questions included on both questionnaires were transferred to the correct data set during processing. Because the state and local questionnaires differ by jurisdiction, students in these schools were not asked all national YRBS questions. Therefore, the total number of students answering each question varied.

### Response Rates, Nonresponse Bias Analyses, and Weighting

Site-level data sets were cleaned and edited for inconsistencies. Missing data were not statistically imputed. A questionnaire failed quality control when <20 responses remained after editing or when it contained the same answer to ≥15 consecutive questions. In 2019, CDC piloted the use of nonresponse bias analysis to determine if a site had data that could be weighted to be representative of its jurisdiction. In previous YRBS cycles, CDC weighted data for any site with an overall response rate (calculated by multiplying school and student response rates) ≥60% (*10*,*11*). For the 2021 YRBS cycle, CDC conducted nonresponse bias analyses for all sites to determine whether data for each site could be weighted to be representative of its jurisdiction. These analyses compared responding and nonresponding schools on school enrollment size (small, medium, or large), a measure of the school’s poverty level (usually the percentage of students eligible for free or reduced-price lunch), and locale type (city, suburban, town, or rural). Analyses also compared responding and nonresponding students by grade and weighted sample and population percentages by grade, sex, and race and ethnicity. If limited statistically significant differences between comparison groups were found, data were weighted to be representative of their respective populations.

A weight calculated as the product of school base weight, student base weight, school nonresponse adjustment factor, student nonresponse adjustment factor, and poststratification adjustment factor based on student sex, grade, and race and ethnicity was attached to each record to adjust for school and student nonresponse in each jurisdiction. The weighted count of students equals the student population in each jurisdiction. A total of 45 states, two tribal governments, three territories, and 28 local school districts, had representative (weighted) data in 2021 (Figures 1 and 2). In 16 states and 19 local school districts, weighted estimates were representative of all students in grades 9–12 attending regular public schools, and in 28 states and nine local school districts, weighted estimates were representative of regular public school students plus students in grades 9–12 in other types of public schools (e.g., alternative or vocational schools).

### Data Availability and Dissemination

A combined data set including national, state, tribal, territorial, and local school district YRBS data (1991–2021) is available from the YRBSS data and documentation website (https://nccd.cdc.gov/Youthonline/App/Default.aspx). Availability of site data depends on survey participation, data quality, and data-sharing policies. Information about YRBSS data is available on the participation maps and history website (https://www.cdc.gov/healthyyouth/data/yrbs/participation.htm). Data requests and other YRBS-related questions can be sent to CDC by using the data request form. (The YRBSS question, comment, and data request form is available at https://www.cdc.gov/healthyyouth/data/yrbs/contact.htm.) Site-level YRBS data collected during 1991–2021 are available through Youth Online (https://nccd.cdc.gov/Youthonline/App/Default.aspx), the YRBS Analysis Tool (https://nccd.cdc.gov/YRBSSanalysis), and YRBS Explorer (https://yrbs-explorer.services.cdc.gov).

## YRBS Response Rates and 2021 Demographic Characteristics

**FIGURE 3:**
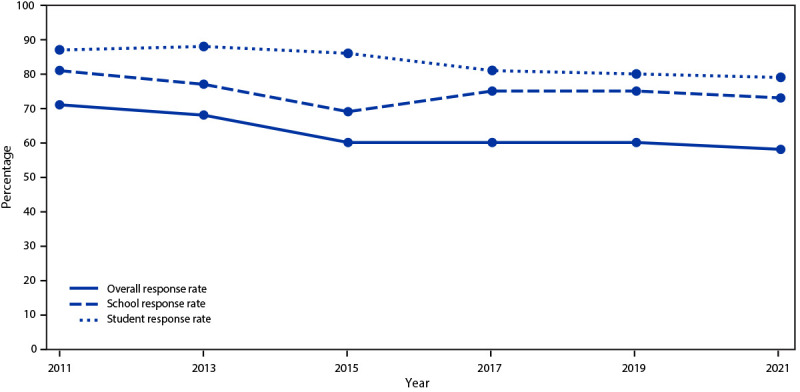
Overall, school, and student response rates for the Youth Risk Behavior Survey, by year of survey — United States, 2011–2021

The 2021 national YRBS overall response rate was 57.5% (Figure 3). This is lower than in previous years and reflects the challenges of conducting a school-based survey during the COVID-19 pandemic. School and student response rates in 2021 (72.7% and 79.1%, respectively) were slightly lower than in the previous two YRBS cycles. Nonresponse bias analyses of the YRBS data found evidence of bias at the school level, but little evidence that this bias significantly affected the national estimates because it was mitigated by weight adjustments based on predictors of nonresponse propensities. YRBS overall response rates have decreased steadily since 2011, with overall response rates in the low 60% range since the 2015 biennial cycle.

**FIGURE 4:**
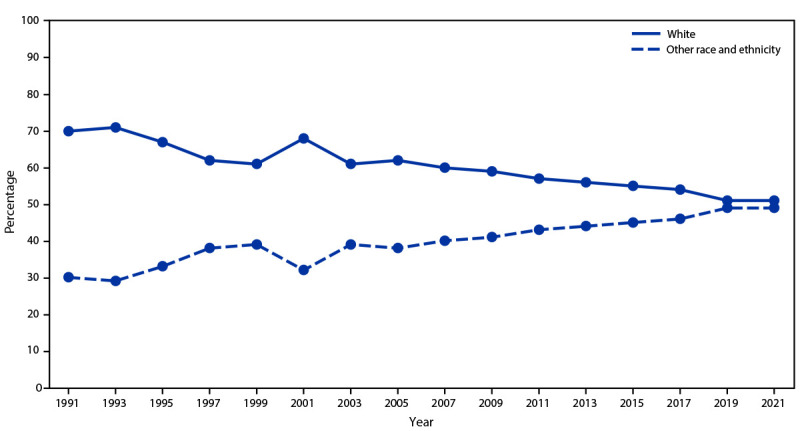
Percentage of students identifying as White or other race and ethnicity,* by year of survey — Youth Risk Behavior Survey, 1991–2021

Data were weighted to match national population proportions. After weighting, approximately half of students were male (51.7%), and percentages of students by grade were as follows: grade 9 (26.6%), grade 10 (25.4%), grade 11 (24.3%), and grade 12 (23.5%) (Table 2). In regard to race and ethnicity, 50.7% of students were White, followed by Hispanic (25.4%), Black (12.1%), multiracial (5.7%), Asian (4.9%), AI/AN (0.7%), and NH/OPI (0.5%). The percentage of students in racial and ethnic groups other than White who participated in the national survey has increased steadily over the past 20 years, from 32% in 2001 to 49% in 2021 (Figure 4).

**TABLE 2:** Student demographic characteristics and response rates — Youth Risk Behavior Survey, United States, 2021

Characteristic	No. (%)
Student sample size*	**17,232 (100)**
**Response rate**	
Schools	(72.7)
Students	(79.1)
Overall	(57.5)
**Sex^†^**	
Female	8,152 (48.3)
Male	8,816 (51.7)
**Race and ethnicity^§,¶^**	
American Indian or Alaska Native	145 (0.7)
Asian	850 (4.9)
Black or African American	2,322 (12.1)
Native Hawaiian or other Pacific Islander	88 (0.5)
White	9,151 (50.7)
Hispanic or Latino	3,244 (25.4)
Multiracial	1,000 (5.7)
Grade**	
9	4,646 (26.6)
10	4,466 (25.4)
11	4,118 (24.3)
12	3,843 (23.5)
**Sexual identity^††^**	
Heterosexual	12,421 (75.5)
Gay or lesbian	520 (3.2)
Bisexual	1,848 (12.1)
Questioning	823 (5.2)
Other	659 (3.9)
**Sexual contact^¶¶^**	
Opposite sex only	4,762 (34.6)
Same sex only	314 (2.4)
Both sexes	744 (6.0)
No sexual contact	7,597 (57.0)

* Among the 17,508 completed questionnaires, 276 failed quality control and were excluded from analysis, resulting in 17,232 usable questionnaires.

^†^ Does not include 264 students who did not indicate sex.

^§^ Persons of Hispanic or Latino (Hispanic) origin might be of any race but are categorized as Hispanic; all racial groups are non-Hispanic.

^¶^ Does not include 432 students who did not indicate race, ethnicity, or both.

** Does not include 23 students who responded, “ungraded other grade” and 136 students who did not indicate a grade.

^††^ Does not include 330 students who responded, “I do not know what this question is asking” and 631 students who did not indicate sexual identity.

^¶¶^ Does not include 3,815 students who did not indicate sex, sex of sexual contacts, or both.

**FIGURE 5:**
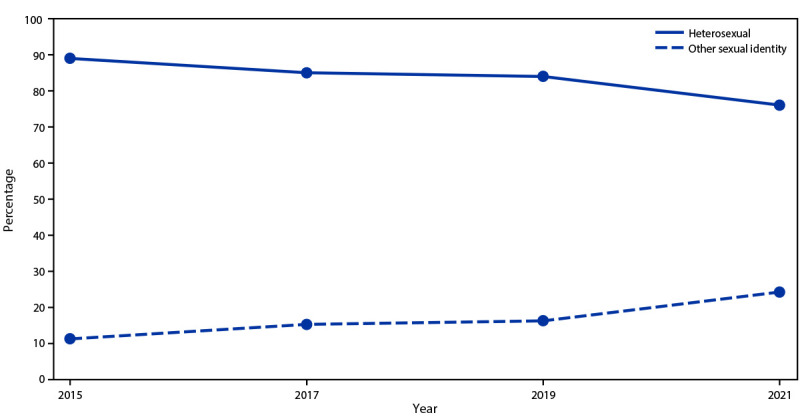
Percentage of students identifying as heterosexual or other sexual identities, by year of survey — Youth Risk Behavior Survey, 2015–2021

In 2021, a total of 75.5% of students self-identified as heterosexual, 3.2% as gay or lesbian, 12.1% as bisexual, 5.2% as questioning, and 3.9% as other (Table 2); 1.8% responded with “I do not know what this question is asking” (data not shown). The percentage of students with a sexual identity other than heterosexual has increased steadily, from 11% in 2015 to 26% in 2021 (Figure 5). Increases in the percentage of LGBQ+ students in YRBSS 2021 might be a result of changes in question wording to include students identifying as questioning, “I am not sure about my sexual identity (questioning),” or other, “I describe my sexual identity in some other way.” In 2021, a total of 57% of students reported no sexual contact during their lives. An estimated 34.6% of students had sexual contact with the opposite sex only, 6.0% with both sexes, and 2.4% with the same sex only.

## Discussion

YRBSS is the largest public health surveillance system in the United States, monitoring multiple health-related behaviors among high school students. The results of the 2021 YRBSS surveys provide the first comparison of youth health behaviors since the onset of the COVID-19 pandemic, using long-term public health surveillance data. Although multiple surveys assessing youth health behaviors have been conducted since March 2020 (*5*,*16*), including ABES, each was limited by small sample sizes, lack of representativeness, or inability to compare findings with those from previous years. YRBS findings can be compared with data from previous YRBS cycles and used in combination with other surveys to provide a robust assessment of youth health needs as communities continue to rebound from the consequences of the pandemic.

Nationally representative data from youths are crucial for identifying health needs and creating evidence-based interventions. The findings of the 2021 YRBS revealed increasing diversity among U.S. high school students. Approximately half of all students identified as being from a racial and ethnic group other than White (49.3%), compared with 48.9% in 2019 and 46.5% in 2017. This shift in youth demographics aligns with U.S. Census projections (*8*) that racial and ethnic populations other than White will account for the majority of all Americans by 2045. In addition, approximately one in four students identified as LGBQ+. Improvements to existing questions and methodology and the introduction of new YRBS questions provide an opportunity to identify needs for an increasingly diverse population of students and address emerging adolescent health issues.

In 2021, YRBS response rates were below 60%, continuing a previously reported decline in YRBS response rates (*11*). Whereas a part of the drop in YRBS participation was attributable to school-level COVID-19 safety precautions, disinformation campaigns targeting YRBSs across the country also contribute to declining YRBS response rates (*17*). Such campaigns misrepresent survey content, data collection procedures, and data utility. Although YRBS data use is at an all-time high, increasing parent refusals for student participation might ultimately prevent a state or locality from obtaining representative data.

New questions featured in the 2021 YRBS on housing instability, exposure to community violence, mental health, and protective factors (parental monitoring and school connectedness) expand the reach of youth health data and address important issues affecting youths. For example, results from the report on school connectedness found approximately 62% of students felt connected to others at school. Although there was variation by race and ethnicity and sexual identity, overall, students who felt connected to others at school had a lower prevalence of all examined risk factors, including poor mental health, prescription opioid misuse, and missing school because of feeling unsafe (*18*). Findings from the report on housing instability indicate that approximately 3% of students experienced housing instability. Students who experienced housing instability were more likely to be LGBQ+ than heterosexual; more likely to be AI/AN, Black, or NH/OPI than White; and more likely to experience sexual or physical violence, persistent feelings of sadness or hopelessness, and suicidal ideation compared with their peers who were stably housed (*19*). Finally, findings from the report on community violence demonstrate that overall, 20% of students witnessed community violence and 3.5% carried a gun. AI/AN, Black, and Hispanic students witnessed more community violence and were more likely to carry a gun compared with their White peers (*20*).

In addition to new questions, prevalence and patterns in health behaviors identified in other reports on longstanding YRBS topics also reinforced the need for specific, tailored public health interventions and resources to improve student health. The report on suicidal thoughts and behaviors indicated an approximately 6 percentage point increase (24% to 30%) from 2019 to 2021 in the prevalence of female students overall who reported seriously considering attempting suicide (*21*). From 2019 to 2021, Black, White, and Hispanic female students experienced increases in prevalence of reporting seriously considering attempting suicide (*21*). Other findings indicated a 3.7 percentage point decrease from 2019 to 2021 in the prevalence of HIV testing and a 5 percentage point decrease in the prevalence of testing for sexually transmitted infections among sexually active students (*22*).

## Limitations

Each report in this supplement includes a limitations section pertaining to that report. In general, YRBSS findings are subject to at least six limitations. First, these data apply only to students in grades 9–12 who attend public and private schools in the United States. Homeschooled students are not included nor are persons who do not attend school; therefore, data are not representative of all persons in this age group. In 2019, approximately 5% of youths aged 14–17 years were not enrolled in school (https://nces.ed.gov/programs/digest/d20/tables/dt20_103.20.asp?current). Second, the extent of underreporting or overreporting of health-related behaviors cannot be determined, although the 2021 survey questions examined demonstrated strong test-retest reliability (*13*,*14*). Third, not all states and local school districts administer the YRBS, and those that did administer it might not have included all standard questions on their YRBS questionnaire; therefore, data from certain questions are not available from all sites. For schools in both the national sample and a state or local sample, the total number of students answering each question varied. Fourth, YRBS data analyses are based on cross-sectional surveys and can only indicate association between variables, not causality. Moreover, the survey is descriptive and not designed to explain the reasons behind any observed trends. Fifth, whereas the national survey historically has been administered during the spring semester, in 2021 it was administered during the fall semester, which might affect comparisons with previous YRBS cycles. Finally, COVID-19 precautions might have reduced school and student participation, although more schools were sampled than in previous cycles to obtain sufficient numbers of students for the desired analyses.

## Conclusion

As students and schools emerge from the COVID-19 pandemic, youth health data are vital to understanding and improving the health of adolescents in the United States. YRBSS remains the best source for quality data at the national, state, tribal, territorial, and local school district levels for monitoring health-related behaviors that contribute to the leading causes of mortality and morbidity among U.S. high school students and that can lead to health problems as adults. Since its inception in 1991, the YRBSS has collected data from approximately 5 million high school students in approximately 2,200 separate surveys. In 2021, in addition to the national data, 45 states, two tribal governments, three territories, and 28 local school districts received data representative of their high school student populations (Figure 1).

Shifts in student demographics can be met with school health programs to help diverse youth populations. To meet the needs of a changing student population, school health programs and policies must also shift to prioritize health equity and the unique needs of racial and ethnic and sexual minority students and consider structural and community-level factors that influence health behaviors and resulting health outcomes.

This overview report describes YRBSS methods for guiding the analyses presented in this *MMWR* supplement. A full description of 2021 YRBS results and downloadable data are available (https://www.cdc.gov/healthyyouth/data/yrbs/index.htm).

## Footnotes

* The standard YRBS questionnaire included 87 questions. YRBS coordinators (located in CDC-funded states, tribes, territories, and local school districts) voted for or against each proposed change, addition, and deletion. Final content of the standard YRBS questionnaire was decided based on the results of this voting process.

† See e.g., 45 C.F.R. part 46.102(l)(2), 21 C.F.R. part 56; 42 U.S.C. §241(d); 5 U.S.C. §552a; 44 U.S.C. §3501 et seq.

§ CDC funds states, tribes, territories, and local school districts, referred to throughout this report as sites, to collect YRBS data. YRBS site-level surveys are conducted among students attending public schools by using samples representative of the state, tribal, territorial, or local school district where they are administered.
